# Feasibility of a hybrid clinical trial for respiratory virus detection in toddlers during the influenza season

**DOI:** 10.1186/s12874-021-01474-9

**Published:** 2021-12-05

**Authors:** Soledad Muñoz-Ramírez, Begoña Escribano-López, Vallivana Rodrigo-Casares, Carlos Vergara-Hernández, Desamparados Gil-Mary, Ignacio Sorribes-Monrabal, María Garcés-Sánchez, María-Jesús Muñoz-Del-Barrio, Ana-María Albors-Fernández, María-Isabel Úbeda-Sansano, María-Victoria Planelles-Cantarino, Ester-María Largo-blanco, Eva Suárez-Vicent, Javier García-Rubio, Patricia Bruijning-Verhagen, Alejandro Orrico-Sánchez, Javier Díez-Domingo

**Affiliations:** 1 Fundación para el Fomento de la Investigación Sanitaria y Biomédica de la Comunitat Valenciana (FISABIO-Public Health), Valencia, Spain; 2 Primary Health Center Malvarrosa, Valencia, Spain; 3 Primary Health Center Serrería II, Valencia, Spain; 4 Primary Health Center Nazaret, Valencia, Spain; 5 Primary Health Center Trafalgar, Valencia, Spain; 6 Primary Health Center L’Eliana, Valencia, Spain; 7 Primary Health Center Paiporta, Valencia, Spain; 8 Primary Health Center Burriana II, Castellón, Spain; 9 Julius Center for Health Sciences and Primary Care, University Medical Center Utrecht, Utrecht, Netherlands

**Keywords:** Acute respiratory infection, Hybrid clinical trials, Influenza, Nasal swab, Parent Collection, electronic diaries, Mobile App Surveillance

## Abstract

**Background:**

Traditional clinical trials are conducted at investigator sites. Participants must visit healthcare facilities several times for the trial procedures. Decentralized clinical trials offer an interesting alternative. They use telemedicine and other technological solutions (apps, monitoring devices or web platforms) to decrease the number of visits to study sites, minimise the impact on daily routine, and decrease geographical barriers for participants. Not much information is available on the use of decentralization in randomized clinical trials with vaccines.

**Methods:**

A hybrid clinical trial may be assisted by parental recording of symptoms using electronic log diaries in combination with home collected nasal swabs. During two influenza seasons, children aged 12 to 35 months with a history of recurrent acute respiratory infections were recruited in 12 primary health centers of the Valencia Region in Spain. Parents completed a symptom diary through an ad hoc mobile app that subsequently assessed whether it was an acute respiratory infection and requested collection of a nasal swab. Feasibility was measured using the percentage of returned electronic diaries and the validity of nasal swabs collected during the influenza season. Respiratory viruses were detected by real-time PCR.

**Results:**

Ninety-nine toddlers were enrolled. Parents completed 10,476 electronic diaries out of the 10,804 requested (97%). The mobile app detected 188 potential acute respiratory infections (ARIs) and requested a nasal swab. In 173 (92%) ARI episodes a swab was taken. 165 (95.4%) of these swabs were collected at home and 144 (87.3%) of them were considered valid for laboratory testing. Overall, 152 (81%) of the ARIs detected in the study had its corresponding valid sample collected.

**Conclusions:**

Hybrid procedures used in this clinical trial with the influenza vaccine in toddlers were considered adequate, as we diagnosed most of the ARI cases on time, and had a valid swab in 81% of the cases. Hybrid clinical trials improve participant adherence to the study procedures and could improve recruitment and quality of life of the participants and the research team by decreasing the number of visits to the investigator site.

This report emphasises that the conduct of hybrid CTs is a valid alternative to traditional CTs with vaccines. This hybrid CT achieved high adherence of participant to the study procedures.

**Trial registration:**

2019–001186-33 (EudraCT).

## Background

Traditional clinical trials (CTs) are conducted at investigator sites. Participants must visit healthcare facilities several times for the trial procedures. The physical location of the center is an important factor in the decision to join a research study [1]. Recruitment and retention continue to be key challenges in randomised CTs [1].

Decentralized CTs use telemedicine and other technological solutions (apps, monitoring devices or web platforms) [2, 3] to decrease the number of visits to study sites, minimise the impact on daily routine, and decrease geographical barriers for participants [1, 4]. Decentralisation allows participants to take part in CTs conducted anywhere [5]. In addition, a pandemic situation (as the one caused by SARS-CoV-2) poses an extra challenge to CTs because most of them might be stopped to avoid infections and virus dissemination at the investigator site.

However, not all CTs may be performed remotely. There are mixed trials, called hybrid CTs that use remote infrastructures in combination with face-to-face procedures performed at investigating sites by qualified staff (chest X-rays, treatment administration, etc.). Hybrid approaches may increase trial flexibility [5].

Traditional CTs with influenza vaccines require additional visits in every possible case of an influenza–like illness (ILI) [EudraCTs: 2016–004904-74, 2013–001231-51, 2011–000758-41]. Procedures such as vaccination, health status examination, recording of ILI symptoms and swabbing are usually performed by health care workers (HCWs) at the investigator site [EudraCTs: 2016–004904-74, 2013–001231-51, 2011–000758-41]. The total number of visits in these CTs could be greatly reduced using hybrid trial procedures, such as self-collected swabs (SCS) or remote ILI symptoms collection.

A recent meta-analysis compared the diagnostic accuracy and validity of SCS when compared to professional-collected swabs in subjects of all age groups with influenza symptoms. Overall sensitivity of SCS was 87% (95% IC: 80–92%) and specificity was 99% (95% CI: 98, 100%) [6]. These figures could introduce a non-differential misclassification bias on the outcome, which in principle should not change the calculation of vaccine effectiveness (understood as a relative measure).

Furthermore, in children, nasal swabs (NS) and nasopharyngeal swabs provided similar results for influenza [6–8]. NS are easier to collect. Parents reported that collection of NS was not difficult [6, 7, 9, 10], and a majority thought that it was not stressful for their children [9]. In addition, parental involvement increases the likelihood of pathogen identification because samples are collected closer to the onset of the acute respiratory infection (ARI) [6, 7, 10, 11].

Respiratory study participants are used to record ILI symptoms in paper diaries [7, 9, 11, 12] for which HCWs perform follow-up through regular phone calls and emails ([13–15], EudraCTs: 2016–004904-74, 2013–001231-51, 2011–000758-41). There is a recent trend to use electronic data collection. Participants entering data at home increase accuracy, and the researcher team has instant, virtual access to data [16]. Acceptance was previously tested in long-term conditions [17–19]. Participant attrition rates ranged from 8.75 and 26% in chronic diseases with long-term follow-up [18]. No similar studies have been conducted in short-term conditions such as, for example, the influenza season.

We are involved in VIGIRA, a hybrid CT which uses electronic log diaries to detect potential ARIs in toddlers, in combination with parent-collected swabs. Parents’ capability to use a mobile app to monitor ARIs was also assessed, as there is not much information available in this field [20]. VIGIRA was ongoing during the two COVID-19 years, so the recruitment was lower than expected. An assessment of the performance of the hybrid CT was done before considering the continuation of the CT.

In this paper we analyse the feasibility of the procedures of a hybrid CT during the influenza season in toddlers.

## Methods

### Study design

VIGIRA is a phase IV multicenter, randomised, double blind, controlled, non-commercial hybrid CT in toddlers to assess the impact of the inactivated quadrivalent influenza vaccine on influenza and other respiratory infections (EudraCT Number: 2019–001186-33).

### Study setting and population

During the 2019/2020 and 2020/2021 influenza seasons, children aged 12 to 35 months with a history of at least 3 ARIs (rhinitis, acute otitis media, pharyngitis, bronchitis, bronchiolitis or pneumonia) in the past 6 months (or 4 in the past year) or 1 severe ARI episode (that required a visit to the emergency room or specialist or hospital admission) in the past year were recruited in 12 primary health centers of the Valencia Region in Spain.

Specifically, we use the APP usage data and the validity of the samples collected by the parents of the 99 children who participated in the first two seasons, making use of all available information. Initially, the study was planned to last for two seasons, reaching a sample size of 400 children (100 per arm and season), but the restrictions promoted by the COVID-19 pandemic made it necessary to modify this estimate and extend the study to a third season (ongoing) to reach the planned sample size. The selection of participants was not random, but by convenience (being a clinical trial): all parents of eligible children attending the primary care centres collaborating in the study were invited to participate.

### Mobile app data collection

From the first study visit to the end of each influenza season, parents used an ad hoc mobile app (VIGIRA, available in the Apple/Play store). Every day, VIGIRA app sent a notification task asking for the presence or absence of 7 symptoms: fever, cough, rhinorrhoea, earache, otorrhea, odynophagia, and shortness of breath or wheezing. Using an algorithm and based on data entered, VIGIRA app discriminated potential ARI episodes and notified parents to collect a swab.

ARIs were defined as: 1) two consecutive days (48 h) with two or more symptoms; or 2) one day with fever and any of the other symptoms and the following day with at least one symptom (with or without fever). Parents who did not perform the required tasks between 48 and 72 h were contacted by the investigating team. Per protocol, subjects were excluded from the study when a correct use of the app was not registered.

### Parent-collected swabs

At the initial visit, HCWs trained parents on NS collection from their children at home. They also had free access to an ad hoc swabbing tutorial video in the VIGIRA app. Each family was provided two NS collection kits containing a FLOQSwab® (recommended for toddlers) and a tube with eNAT™ transport medium (able to stabilise viruses at room temperature for 28 days). Swabbing was done at home or at the investigator site if parents asked for help. Parents brought the samples to the health center at their best convenience, and they were regularly sent to the laboratory. During the SARS-CoV-2 pandemic and the lockdown, a courier service collected the samples from subjects’ homes.

### Laboratory procedures

NS were tested by real-time reverse transcription-PCR (RT-PCR) for 9 different viruses (in﻿fluenza virus A y B, coronavirus, metapneumovirus, bocavirus, adenovirus, respiratory syncytial virus, parainfluenza virus, rhino/enterovirus and SARS-CoV-2) at a central virology laboratory at Fundación para el Fomento de la Investigación Sanitaria y Biomédica de la Comunitat Valenciana (FISABIO-Public Health) following World Health Organisation (WHO) protocols [21]. Samples were considered valid based on their threshold cycle (Ct) value (Ct ≤ 35) for the human cellular control RNAse-P.

### Statistical analysis

Adherence to VIGIRA app use was assessed based on the proportion of all diaries that were fully filled out and the proportion of all NS requested that were collected. The proportion of valid home-collected NS was also presented.

## Results

Ninety-nine subjects were recruited during two influenza seasons. 52 (52.5%) males and 47 (47.5%) females. Data collection was performed from November 13th 2019 to April 12th 2020 during the first season and from November 3th 2020 to March 15th 2021 during the second season. The median follow-up was 124 days (IQR: 65–151) and 103 (IQR: 90–117) respectively.

Three subjects were excluded from the analysis because abnormal data were entered of the app, including: 1) systematic introduction of several symptoms with maximum severity every day of the study, after pediatrician assessment; 2) date mismatch between symptom registration and sample collection; and 3) failure to collect more than 50% of the samples requested despite reminders by the study coordinator.

During the first and the second season, parents filled out 5652 and 4824 diarie, i.e. 95.5 and 98.7% of all diaries requested respectively. Of the 188 potential ARI episodes that required sampling, 173 (92%) swabs were collected. HCWs took 8 (4.6%) mainly because parents attended the clinic for the assessment of the disease. 14 (8.5%) NS collected were not received at the laboratory (6 from the first season and 8 from the second). From them, 7 samples were lost at home, 3 samples could not be sent due to the pandemic emergency lockdown and 4 samples were lost at the primary health centre before sending them to the laboratory. 7 collected samples were invalid.

Overall, 144 (87.3%) NS collected at home were valid (Table 1). A valid sample was collected in 152 of the 188 ARI (80.9%).

**Table 1:** Description of the participants and the development of the HCT during the two seasons. Samples with a Ct value < 35 were considered valid

	Total	First	Second
Sex, n (%)			
Males	52	31 (59.6%)	21 (40.3%)
Females	47	21 (44.7%)	26 (55.3%)
Follow-up (days), median (IQR)	227	124 (65-151)	103 (90-117)
Diaries Requested, n	10804	5918	4886
Diaries Completed, n (%)	10476 (97%)	5652 (95.5%)	4824 (98.7%)
Detected ARI	188	119	69
Samples collected, n (%)	173 (92%)	105 (88.2%)	68 (98.6%)
By HCW	8 (4.6%)	4 (3.8%)	4 (5.9%)
By parents	165 (95.4%)	101 (96.2%)	64 (94.1%)
Not received at lab	14 (8.5%)	6 (5.9%)	8 (12.5%)
Valid home NS	144 (87.3%)	88 (87.1%)	56 (87.5%)
Invalid home NS	7 (4.2%)	7 (6.9%)	0

As shown in Table 1, there was an improvement in the 2nd season in the quality of the swabs, as all samples taken at home contained enough biological material to be analysed. However, there was still room for improvement in the handling of the samples. We are reviewing our procedure manual to lessen the number of samples not reaching the lab.

In order to monitor parents’ compliance with the trial requirements and to solve VIGIRA app problems, the coordinating team reviewed use of the VIGIRA app through the web site for a total of 1826 min and made 120 phone calls: 18.4 min/subject and 1.2 phone calls/subject during the first season.

## Discussion

In this study, the feasibility of a hybrid CT with influenza vaccine in toddlers was assessed. Use of electronic app diaries for symptom collection and parent-collected swabs had enough quality to continue the trial for a third season.

Previous epidemiological studies have used parental recording of symptoms and home-collected swabs. In a feasibility study of a birth cohort intended to assess acute infections using paper-based symptom diaries and parental collection of NS, adherence to both procedures was 77.3 and 62.7% respectively [9]. It was concluded that the detection rate of respiratory viruses (64.3%) was similar to that achieved with throat swabs collected in a governmental ARI surveillance project (ARI Surveillance in Lower Saxony) [9]. In another study on the cost of community-managed viral respiratory illnesses in a cohort of healthy preschool children, 487 ARIs (67%) had at least one sample and a paper diary available, 41 (6%) had a diary returned but no sample, 56 (8%) had one sample returned but no diary available, and 146 (20%) had neither of the two [12]. Among the 523 ARIs with a diary returned, 24.1% had no virus identified and 7.6% had no specimen [12]. Another community-based cohort study wanted to know if parents were more receptive to participate in a study of respiratory infections when samples were taken at home, thus avoiding travel to the health centre [7]. Adherence to the daily symptom diary was 82.5%, and sampling adherence was 74.4% [7]. Objectives, ARI definitions, eligibility criteria, and sample collection criteria were very heterogeneous among all of these studies. In spite of the differences among studies, adherence to symptom collection in paper diaries ranged from 73 and 82.5%, and sampling adherence ranged from 62 to 75% [7, 9, 12].

The VIGIRA app has been shown to be an intuitive and user-friendly interface that requires minimal training and achieves an excellent participant adherence in terms of completed diaries (97%). Moreover, completed diaries cannot be lost because they appear instantly in the investigator’s website. Reaching a significant number of tested samples is always the cornerstone in respiratory studies. The proportion of valid samples as compared to the total potential ARI episodes detected in the VIGIRA study was 80.9%. Even if all the samples collected in the abovementioned epidemiological studies are considered valid, the percentage reached with VIGIRA was higher.

VIGIRA cannot be fully compared to other CTs because this is, to our knowledge, the first CT using this methodology. A comparison of our percentage of valid samples with recent traditional CTs with the influenza vaccine in toddlers has not been possible because we do not have access to that information. In a traditional randomized CT on influenza, HCWs perform ARI surveillance throughout the influenza season. For each potential ARI episode, one successful surveillance contact and one sampling visit are needed [EudraCTs: 2016–004904-74, 2013–001231-51, 2011–000758-41]. HCWs record symptoms and perform ARI surveillance until the ARI process ends. These procedures are very time and resource consuming. According to VIGIRA hybrid model, the time consumed to record the 10,476 (97%) diaries completed and to detect the 188 potential ARIs was minimal because these tasks were done automatically by the VIGIRA app. In addition, no HCWs were required for 165 NS (95.4%) because they were collected at home.

There are some limitations regarding hybrid procedures. Even after several retraining, three subjects (3%) in the first season did not understand to use the VIGIRA app. Erroneous and altered information was entered into the app, and tasks were not carried out properly. These subjects were from two investigating sites that covered a low sociocultural area. Another limitation is that our hybrid procedures included participants who met a number of requirements: they should have a smartphone, Internet access, digital skills, and knowledge of the Spanish language.

There is room for improvement since no sample was available for 15 (8%) possible ARIs despite several calls and task reminders. 14 samples (8.5%) collected in both seasons did not arrive to the laboratory.

Although travel was largely avoided, even in a hybrid CT some study visits are required. Parents and subjects mustattend the investigating site at least once for signing the informed consent and for vaccination.

The recent public health crisis has shown that establishment of a decentralised methodology is becoming increasingly necessary, but there are many barriers to overcome in hybrid CT. VIGIRA first and second seasons have been a pilot study with a low number of subjects recruited intended to test the hybrid CT procedures. Unlike many other CTs, the VIGIRA study was not discontinued due to SARS-CoV-2 pandemic. Guidelines to be used as a reference for implementing this type of CT are needed. New projects are being developed, such as the H2020-IMI Trials@Home [22], to achieve implementation of decentralized CTs and hybrid CTs in standard practice.

## Conclusion

This report emphasises that the conduct of hybrid CTs is a valid alternative to traditional CTs with vaccines. This hybrid CT achieved high adherence of participant to the study procedures. Hybrid CTs could improve recruitment and quality of life of the participants and the investigator team by decreasing the number of visits to the investigator site.

## Data Availability

The datasets used and analysed during the current study are available from the corresponding author on reasonable request. This article is co-financed by Fondo Europeo de Desarrollo Regional (FEDER).

## Abbreviations

ARI: Acute respiratory infection
CTs: Clinical trials
HCWs: Health care workers
ILI: Influenza–like illness
NS: Nasal swabs
SCS: Self-collected swabs
Ct: Threshold cycle
