# Correction: Feasibility of a hybrid clinical trial for respiratory virus detection in toddlers during the influenza season

**DOI:** 10.1186/s12874-024-02281-8

**Published:** 2024-07-19

**Authors:** Soledad Munoz‑Ramirez, Begona Escribano‑Lopez, Vallivana Rodrigo‑Casares, Carlos Vergara‑Hernandez, Desamparados Gil‑Mary, Ignacio Sorribes‑Monrabal, Maria Garces‑Sanchez, Maria‑Jesus Munoz‑Del‑Barrio, Ana‑Maria Albors‑Fernandez, Maria‑Isabel Ubeda‑Sansano, Maria‑Victoria Planelles‑Cantarino, Ester‑Maria Largo‑blanco, Eva Suarez‑Vicent, Javier Garcia‑Rubio, Patricia Bruijning‑Verhagen, Alejandro Orrico‑Sanchez, Javier Diez‑Domingo

**Affiliations:** 1 Fundación para el Fomento de la Investigación Sanitaria y Biomédica de laComunitat Valenciana (FISABIO-Public Health), Valencia, Spain; 2 Primary Health Center Malvarrosa, Valencia, Spain; 3 Primary Health Center Serrería II, Valencia, Spain; 4 Primary Health Center Nazaret, Valencia, Spain; 5 Primary Health Center Trafalgar, Valencia, Spain; 6 Primary Health Center L’Eliana, Valencia, Spain; 7 Primary Health Center Paiporta, Valencia, Spain; 8 Primary Health Center Burriana II, Castellón, Spain; 9 Julius Center for Health Sciences and Primary Care, University Medical Center Utrecht, Utrecht, Netherlands

**Correction: BMC Med Res Methodol 21**,** 273 (2021)**

10.1186/s12874-021-01474-9

Following publication of the original article [1], the authors reported an error located in the funding section. The following statement “This article is co-financed by Fondo Europeo de Desarrollo Regional (FEDER)” was missing.

The original article [1] has been updated.
